# Fault detection in electrical power systems using attention-GRU-based fault classifier (AGFC-Net)

**DOI:** 10.1038/s41598-025-06493-w

**Published:** 2025-07-06

**Authors:** Deepen Khandelwal, Prateek Anand, Mayukh Ray, Sangeetha R. G.

**Affiliations:** School of Electronics Engineering, Vellore Institute of Technology, Chennai, 600127 Tamil Nadu India

**Keywords:** Electrical Circuit, AGFC-Net, GRU, Attention Mechanism, Engineering, Electrical and electronic engineering

## Abstract

Fault detection is essential in guaranteeing the reliability, security, and productivity of contemporary technological and industrial systems. Faults that go unnoticed may result in disastrous failures as well as prohibitive downtimes in industries as varied as healthcare, manufacturing, and autonomous functioning. Conventional fault detection technologies tend to possess low accuracy rates, weak feature extraction, as well as limitations in generalizability across variegated faults. To overcome these shortcomings, this paper puts forward an Attention-GRU-Based Fault Classifier (AGFC-Net), which employs a sophisticated attention mechanism for improved feature extraction and correlation learning. Through the fusion of attention layers with Gated Recurrent Units (GRU), AGFC-Net is able to focus on key fault features, learn temporal dependencies, and provide better classification performance even under noisy conditions. Experimental results show that AGFC-Net attains a fault detection accuracy of 99.52%, better than conventional machine learning and deep learning algorithms. The suggested method presents a stronger, adaptive, and scalable solution for autonomous fault diagnosis, opening the door to intelligent and trustworthy fault detection systems in future power grids and industrial applications.

## Introduction

A transmission line is a critical component of an electrical power system, acting as the conduit through which electrical energy is transferred from generating stations to wider distribution networks. With the global rise in electricity demand over recent decades, maintaining stable and reliable transmission lines has become increasingly essential. However, transmission lines are prone to various types of faults caused by environmental conditions such as lightning, wind, and storms, as well as by equipment aging, insulation failure, or external mechanical impacts. If these faults are not detected and addressed promptly, they can result in large-scale power outages, damaging infrastructure and disrupting grid stability. Therefore, accurate and timely fault identification is a crucial requirement for the secure operation of modern power systems.

The process of detecting and classifying faults in electrical transmission lines is inherently complex due to the dynamic nature of the power system and the interactions among its numerous components. Transmission lines operate within vast, interconnected electrical networks that include generators, transformers, circuit breakers, and protective relays. These elements continuously interact with one another, making it essential that fault detection systems not only identify the occurrence of faults but also classify them accurately according to their type and origin. Faults in transmission systems can manifest in different forms, such as single line-to-ground faults, line-to-line faults, double line-to-ground faults, or symmetrical three-phase faults. Each type has unique electrical characteristics and implications, requiring tailored detection and protection strategies to minimize equipment damage and restore system stability.

In modern power systems, fault detection forms a foundational element of the protection mechanism. Upon the occurrence of a fault, the protection scheme must quickly and reliably detect the event, determine the nature and location of the fault, and trigger the appropriate control action–typically by isolating the faulty section using circuit breakers and relays. Traditional fault detection approaches have relied heavily on hardware-based solutions like electromechanical relays and threshold-based mechanisms, which, despite their simplicity, often lack adaptability and fail to meet the accuracy and speed requirements of today’s dynamic grids. With the increasing complexity of power systems, such conventional approaches have shown limitations in response time, feature generalization, and adaptability to various fault scenarios, particularly in systems with renewable energy sources and decentralized architectures. Recent developments in sensor technologies and data acquisition systems have resulted in the installation of intelligent monitoring equipment across the power grid. Such devices continuously monitor a broad variety of electrical parameters, such as voltage, current, frequency, and phase angle, and offer high volumes of high-resolution data. Utilization of this data through advanced computational methods has been a major driver for creating more precise and adaptive fault detection models. In particular, model-based techniques are increasingly being used to surmount the inflexibility of conventional rule-based systems.

Machine learning (ML) and pattern recognition algorithms have proven to be strong contenders for fault detection and diagnosis. By learning from past or simulated fault data, these systems are able to automatically develop intricate patterns of association between system measurements and fault states. For example, support vector machines (SVM), artificial neural networks (ANN), and decision tree algorithms have been used to classify faults in three-phase transmission lines successfully. These models are capable of identifying non-linear patterns and enhancing generalization through supervised learning, thus improving their performance under fluctuating system conditions.

Nevertheless, despite classical ML models providing superior flexibility and prediction accuracy compared to previous threshold-based techniques, they tend to rely on hand-designed features and are incapable of describing the temporal evolution of electrical signals. This is especially challenging in fault cases where electrical parameters change over time in subtle but informative patterns. Furthermore, ML models might not be as robust as required for real-time use, particularly in the presence of noise and operating variations. These challenges have motivated the use of deep learning techniques, which can automatically learn relevant feature representations from raw input data without requiring cumbersome preprocessing.

Some of the deep learning models, including convolutional neural networks (CNNs), long short-term memory (LSTM) networks, and hybrid models, have been put forward for electrical fault diagnosis. These models can learn intricate spatial and temporal relationships within the data. Although effective, some models continue to have difficulty in balancing model complexity, interpretability, and computational expense. In addition, most models do not have mechanisms to concentrate on the most informative regions of the input data, resulting in lower efficiency and vulnerability to irrelevant information.

**Fig. 1:**
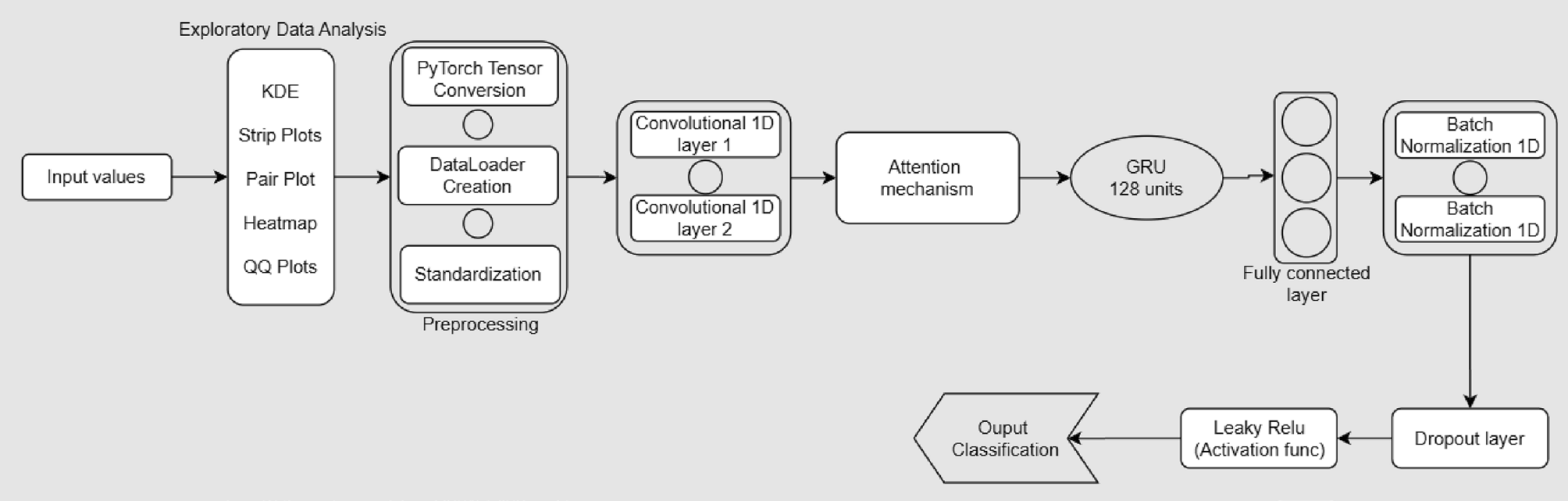
AGFC-Net.

To overcome these shortcomings, this paper proposes an Attention-GRU-Based Fault Classifier (AGFC-Net), a deep learning model that integrates the advantages of convolutional layers, attention mechanisms, and gated recurrent units (GRUs) as shown in Fig. 1. Convolutional layers are utilized to derive spatial features from multivariate time-series like voltage and current readings. The attention mechanism receives these features, which selectively attends to the most pertinent signal sections to minimize noise effects and maximize interpretability. The GRU layers are used later to detect temporal dependencies, enabling the model to comprehend how electrical parameters change with time during fault conditions. This blend helps AGFC-Net attain increased classification performance and retain computational efficiency and noise robustness. The dataset employed in this study comprises about 12,000 annotated samples symbolizing both faulty and normal operating states of an emulated three-phase transmission system. Each sample includes six features: line voltages (Va, Vb, Vc) and line currents (Ia, Ib, Ic), captured under different fault types and conditions. The defects range over all combinations of phase fault possibilities and encompass differences in fault location and impedance, offering a wide and all-inclusive training set. This annotated dataset is ideal for supervised learning since the model can learn discriminative features that distinguish normal operation from different types of faults.

The primary goal of this study is to create a strong, precise, and scalable model that can perform real-time fault detection and classification in power transmission lines. Through the incorporation of attention-based learning and GRU architecture, AGFC-Net aims to address the issues of conventional machine learning and deep learning models. The long-term aim is to be able to contribute to the development of intelligent fault detection systems not only capable of recognizing complex fault patterns but also appropriate for implementation within contemporary power grids requiring real-time monitoring and decision-making.

## Literature survey

Saberia et al.^1^ contrasted the support vector machine (SVM) and artificial neural networks (ANN) for the detection of faults in centrifugal pumps. They were interested in various kernels used in SVM, such as Gaussian and linear kernels, and observed that SVM outperformed ANN, particularly under noisy conditions. Noise had little effect on SVM’s capacity to classify faults correctly, thus, it is a more robust approach for real-world applications. However, ANN was more vulnerable to noise, which had a negative effect on its performance.Zhao et al.^2^ also developed a Condition-Based Monitoring (CBM) system based on a Dempster–Shafer-based Genetic Backpropagation (DGBB) model and ANN for bearing failure diagnosis. The system was able to detect faults successfully by mimicking vibration patterns and comparing them with experimental sensor data. The model showed high classification accuracy and was therefore an efficient model for monitoring the health of industrial machinery. The sensitivity of the model to changing fault conditions was also one of the major strengths.Yang^3^ proposed the application of a recursive high-order parameter neural network (RHPNN) for the classification of faults in analogue integrated circuits, specifically for open- and short-circuit faults. The model tackled parametric variations due to faults and greatly improved detection accuracy over conventional methods, demonstrating the ability of neural networks to deal with dynamic circuit faults.Grimaldi and Mariani^4^ used ANN in On-Board Diagnostics (OBD) of automotive engines to improve fault detection. Their application successfully detected faults in engine parts from sensor measurements such as temperature, pressure, and exhaust emissions. Using ANN helped in real-time detection and diagnosis, making it ideal for applications where there is a need to quickly identify faults for maintenance and safety purposes.Yousaf et al.^5^ created a hybrid fault detection model using Long Short-Term Memory (LSTM) networks and Discrete Wavelet Transform (DWT) for the detection of faults in high voltage direct current (HVDC) systems. The proposed model was found to have 99.04% accuracy in fault detection while being robust against other external faults. This model particularly performed well under dynamic operations and had excellent generalization potential for real-time detection, which made it applicable for large-scale power grid systems.B. Samanta et al.^6^ have compared the classification accuracy of SVM and ANNs in gear fault detection. Vibration signals from rotating machines with healthy and faulty gears were utilized to create features for classification. ANN with derivative/integral preprocessing achieved a test accuracy of 97.92%. Nevertheless, SVM performed better than ANN in most cases, especially when combined with GA-based feature selection and parameter tuning. Though ANN proved more accurate in certain instances, SVM showed reduced training times and superior overall performance.Andrade et al. citeAndrade2021 implemented artificial neural networks based on non-linear autoregressive exogenous (NARX) for fault detection in pneumatic systems. The method proved to be effective in classifying faults with a minimal computational time as opposed to traditional techniques. The research highlighted ANN-based methods’ flexibility towards intricate systems involving numerous interdependent components. Je-Gal et al.^8^ introduced a time-frequency feature fusion approach to identify faults in marine engines. The method fused wavelet transforms with deep learning algorithms to extract useful features from vibration signals. The approach was highly accurate in fault identification even under low sampling rates and noisy signals, demonstrating its promise for maritime use where the data bandwidth is restricted. Aherwar^9^ presented a review of fault detection in gearboxes using vibration analysis in time, frequency, and time-frequency domains. The article emphasized the utility of AI methodologies, such as ANN, to enhance fault detection accuracy. The AI-based methodologies were shown to be more computationally efficient and appropriate for real-time monitoring based on their low computational cost. Ge et al.^10^ used Support Vector Machine (SVM) for fault detection in sheet metal stamping processes. The model was able to classify faults like misalignment and overloading, even with a small amount of training data. SVM performed better than ANN, and hence it is a potential candidate for industrial fault detection applications where data is limited. Puig et al.^11^ proposed a passive fault detection technique based on Group Method of Data Handling Neural Networks (GMDHNN) and constraint satisfaction algorithms. The technique was centered on fault detection without needing active testing or intervention in the system. It demonstrated better accuracy in highly uncertain complex systems than conventional methods. Shi et al.^12^ used wavelet transforms and ANN for damage detection in civil engineering structures. Through vibration data analysis, their model obtained high training accuracy (99.52%). This hybrid method effectively integrated wavelet-based feature extraction with ANN classification and applied it to real-time infrastructure health monitoring. Efatinasab et al.^13^ suggested Bayesian Neural Networks (BNN) for fault zone prediction in smart grids. The model’s capability to model input uncertainty minimized false alarms and enhanced prediction accuracy. This work proved that uncertainty modeling can improve reliability when combined with neural networks in uncertain environments like power grids. Jamil et al.^14^ proposed a fault detection system for power transmission lines based on an ANN. The method successfully identified and classified the faults, irrespective of fault location or impedance. The system showed high classification accuracy under diverse conditions, enhancing both speed and reliability in transmission systems. Yadav and Dash^15^ surveyed the use of ANN in transmission line protection, highlighting the advantages of employing ANN for fault detection and classification. The study concluded that ANN models are efficient and effective at dealing with complex fault structures and classifying faults in real time, improving the robustness and efficiency of power grid protection schemes. Veerasamy et al.^16^ proposed an LSTM-based approach for the detection of high impedance faults (HIF) in power systems with PV integration. Their approach detected with 92.42% accuracy and proved to be robust against noisy and complex input data, making it pertinent in the context of smart grid systems with renewable energy integration. Mohanty et al.^17^ used the Cumulative Sum (CUSUM) technique for power system fault detection. The technique improved noise resistance and frequency deviation robustness, making it more applicable in high-voltage conditions with signal quality issues. The research showed that the method improved the accuracy of fault detection. Silva et al.^18^ integrated the wavelet transform with ANN for fault detection in transmission lines. Their approach achieved perfect classification accuracy in simulations and performed well with real-world data. This fusion of wavelet-based feature extraction and ANN classification proved highly effective for real-time fault detection in power distribution systems. An algorithm for fault classification with a combination of Discrete Wavelet Transform (DWT) and Back-Propagation Neural Networks (BPNN) was presented by C. Pothisarn and A. Ngaopitakkul^19^. Fault signals were simulated with ATP/EMTP, and Daubechies4 was employed as the mother wavelet. Components in the first decomposition scale of high frequency were utilized for classification. The technique classified faults with varying types, locations, and inception angles with an accuracy of more than 97.22% , which shows greater efficiency and accuracy compared to conventional techniques. Yong Deng^20^ introduced a better diagnostic approach for analog circuits based on a hierarchical Levenberg-Marquardt (LM) Discrete Volterra Series (DVS) algorithm with a condensed closest neighbor (CNN) classifier, which is referred to as IDVS-CNN. The approach made DVS parameter calculation simpler through hierarchical design and Bayesian information criteria. The model obtained macro and micro F1 scores of 0.903 and 0.894, respectively, with improved fault identification ability and reduced computational complexity. Lamya Gabera^21^ suggested a fault detection model for digital VLSI circuits based on deep learning, i.e., a Stacked Sparse Autoencoder (SSAE). The process included test pattern generation, feature reduction, and classification through a softmax layer. The model was evaluated on eight ISCAS’85 benchmark circuits with a maximum fault coverage of 99.2% and a validation accuracy of up to 99.7%, demonstrating its efficacy in detecting stuck-at faults. Papia Ray et al.^22^ introduced an SVM-based approach for fault classification of long transmission lines with distance estimation, optimized by Particle Swarm Optimization. Energy and entropy features were extracted by Wavelet Packet Transform (WPT), and then feature selection and normalization were applied. The model performed 99.21% accuracy on a 400 kV, 300 km transmission line with 10 short-circuit fault types, and fault distance error less than 0.21%. Applied to a TCSC system, accuracy was 98.36% with an error of around 0.29%, justifying the efficacy of the model in fault detection of a power system. Fouad Suliman^23^ proposed this research that investigates fault identification in photovoltaic (PV) systems based on Support Vector Machines (SVMs) and Extreme Gradient Boosting (XGBoost) optimized with the Bees Algorithm (BA) and Particle Swarm Optimization (PSO). A small PV array was employed to replicate real faults, such as line-to-line and open-circuit faults. BA remarkably improved classifier accuracy, of which BA-XGBoost had 87.56% accuracy and BA-SVM 70.79%, both of which outperformed models based on PSO. The study emphasizes the efficacy of BA in enhancing fault classification accuracy and its applicability for wider use in intelligent fault detection systems in various applications encompassing renewable energy and machine learning integration. AHMED SAMI ALHANAF^24^ developed This work emphasizes the usage of deep learning models–CNN, LSTM, and CNN-LSTM hybrid–for fault detection, classification, and location in smart grids with renewable integration. Based on voltage and current signals, these models exhibited strong performance on IEEE 6-bus and 9-bus systems in the presence of distributed generators (DGs) and network topology variations. The models proved to be more accurate than conventional techniques. Other works proposed DRNNs and CNNs for fault prediction from PMU measurements and attained high accuracy (even 99.92%), albeit a few of them were without fault location capability. Though with robust results, most of them need to be further tested on varied grid configurations and DG environments. Ting Huang^25^ This work proposes a deep learning-based fault diagnosis technique that both integrates feature extraction and fault occurring time delays effectively. The technique couples sliding window processing with a hybrid CNN-LSTM architecture. Sliding windows convert multivariate time series (MTS) data into samples that preserve temporal and feature information. CNN layers automatically perform feature extraction, while LSTM layers preserve temporal relationships and delays. When applied to the Tennessee Eastman chemical process, the new approach outperforms the other methods with higher predictive accuracy and better noise robustness. A comparative study of five popular methods supports its improved performance, demonstrating its suitability for fault diagnosis of complex industrial processes. Xinming Li^26^ The Energy-Driven Graph Neural OOD (EGN-OOD) detector addresses the challenge of out-of-distribution (OOD) detection in intelligent fault diagnosis for construction machinery. By combining graph neural networks with energy-based models, it effectively captures complex fault relationships. Sensor-acquired vibration data is transformed into graph representations using the Maximal Information Coefficient, enabling the modeling of nonlinear fault interactions. Xinming Li^27^ The GCI-ODG framework addresses the challenge of distribution shifts in intelligent fault diagnosis for wind turbines. Leveraging Graph Causal Intervention (GCI), it enhances out-of-distribution (OOD) generalization by capturing both local and global patterns through a hierarchical graph representation of multi-condition time-series data. An adaptive expert ensemble mechanism enables dynamic feature extraction using pseudo-environment labels, improving robustness without explicit environmental data. Additionally, causal inference techniques such as backdoor adjustment isolate stable, environment-invariant features, reducing spurious correlations.

## Methodology

The AGFC-Net model is designed for the complex problem of fault detection in electrical power systems by making use of multiple advanced techniques. The first phase of the model is composed of two convolutional layers, conv1 and conv2, which aim to extract crucial spatial features from the input data, which will be composed of voltage and current measurements from several phases of the transmission lines. These convolutional layers learn directly relevant patterns from raw data; these contain vital characteristics of a system in fault due to variations in voltage and current. In addition, after every convolutional layer, the process includes applying batch normalization to help standardize activations while promoting a smooth, fast convergence speed in training.

Incorporation of the attention mechanism further enhances the model by enabling it to focus its attention on the most important features. This calculates attention weights using a linear transformation followed by a softmax operation that represents scaling critical information and filtering out the noise. It is very effective in fault detectiowell-scaled because it allows the model to filter out those pieces of information that are not useful and place its focus on the highly indicative features of system faults.

After the convolutional and attention layers, the model applies a GRU layer to catch sequential dependencies and temporal patterns. Electrical faults tend to be dynamic in nature, and fault detection in such cases is dependent on the model’s ability to understand how certain patterns change over time. The GRU layer allows the model to monitor these changes and identify complex time-dependent fault patterns that other models might not detect. The update and reset gates in the GRU allow the model to learn what parts of the data are important for each time step, which makes it more sensitive to subtle variations in the behavior of the system.

To prevent overfitting and to generalize better to unseen data, regularization techniques will apply dropout and batch normalization throughout the network. Dropout is applied in training, where it disables a proportion of neurons at random, forcing the model to learn more robust features and not rely too heavily on any single neuron. Batch normalization, as mentioned earlier, makes sure that the activations within the network are well scaled and centered, further improving the model’s ability to generalize.

The output layer of the model finally uses a sigmoid activation function to convert the output into a probability between 0 and 1, indicating the likelihood of a fault occurring. This is a binary classification type of approach that allows the model to classify the system in both normal and faulty conditions.

These combined features allow the model, AGFC-Net, to provide very high accuracy in fault detection because it includes convolutional layers for feature extraction, attention for focusing on the relevant data, GRU layers for capturing the temporal dependencies, and regularization techniques for robustness. Furthermore, this model has much faster processing times and can therefore be applied to real-time applications for fast fault detection and response to faults in modern electrical power grids. The integration of these techniques results in a model that significantly outperforms traditional fault detection methods in terms of both accuracy and efficiency as shown in Table 1.

The proposed Attention-Guided Feature Compression Network (AGFC-Net) introduces a novel architectural synergy that emphasizes efficiency, interpretability, and robustness, despite previous studies exploring the integration of convolutional neural networks (CNNs), gated recurrent units (GRUs), and attention mechanisms for time-series classification and fault diagnosis. Before temporal modeling with a GRU, localized temporal features are extracted using 1D convolutional layers in AGFC-Net. These features are then further tuned using a lightweight attention method applied at the feature level. The network may prioritize salient feature representations early in the pipeline thanks to this attention-guided compression, which lessens the recurrent layer’s learning load and enhances the model’s overall focus.To improve regularization and reduce overfitting, the architecture additionally includes batch normalization and dropout following each convolutional level. A binary classification output is the result of the successive dimensionality reduction intended for the fully linked layers. AGFC-Net, in contrast to traditional CNN-GRU-Attention models, has an emphasis on a simple yet efficient design that is suited for real-time fault detection scenarios in environments with limited resources. The main innovation of AGFC-Net is this simplified combination of temporal modeling and attention-guided feature compression.

## Experimental setup

To evaluate the performance of the AGFC-Net model (Fig. 1), we conduct experiments on the pre-processed dataset. The model is trained using a subset of the data and tested on an independent validation set. The following steps are followed during the experiments:

### Training and validation split

The dataset is split into training (70%), validation (15%), and test (15%) sets. The training set is used to train the model, while the validation set is used to tune hyperparameters and prevent overfitting.

### Model evaluation metrics

The model’s performance is evaluated using standard metrics, including accuracy, precision, recall, and F1-score. These metrics are essential for assessing the model’s ability to detect faults accurately and distinguish between normal and faulty conditions.

### Comparison with baseline models

The AGFC-Net model is compared to traditional fault detection methods, such as Support Vector Machines (SVM) and Artificial Neural Networks (ANN). The comparison focuses on both accuracy and processing time, as the ability to detect faults in real-time is crucial for power system operation.

### Training time and convergence

The training time and convergence speed of the AGFC-Net model are also evaluated. The batch normalization and dropout techniques are specifically assessed for their impact on training efficiency and the model’s ability to converge quickly.

### Real-time application testing

To simulate real-time fault detection, the trained model is tested on streaming data representing a power system in operation. The goal is to assess how quickly and accurately the AGFC-Net model can detect faults in a dynamic, real-world environment.

**Table 1:** Comparison of AGFC-Net with existing models.

Author	Model	Accuracy (%)
Yousaf^5^	LSTM and DWT	99.04
Samanta^6^	SVM and ANNs	97.92
Veerasamy^16^	LSTM	92.42
Ngaopitakkul^19^	ATP/EMTP and Daubechies4	97.22
Lamya Gabera^21^	SSAE	99.21
**Proposed model**	AGFC_net	**99.52**

## Data collection

**Definition**

The dataset for this study, which consists of readings of line voltages (Va, Vb, Vc) and line currents (Ia, Ib, Ic) both under normal and fault conditions in a power system. It contains 12,000 labeled data points for the target variable Output (S) referring to the status of the system as normal or faulty. To facilitate fault analysis, a simulated power system was constructed using MATLAB. The system architecture includes four 11 kV generators, arranged such that two generators are placed at each end of the transmission line. Intermediate transformers are incorporated to replicate realistic power transmission conditions and enable the study of fault behaviour at the line’s midpoint. The system is evaluated under both steady-state (normal) operation and various fault scenarios. During these simulations, line voltage and current measurements are recorded at the transmission line’s output terminal. This process yields a substantial collection of labeled time-series data, encompassing both normal and faulty operating conditions, suitable for training and evaluating fault classification models. Preprocessing steps include

- **Handling missing values:** The missing values will be checked in the dataset. Then, missing values will be handled either by imputation or deletion of related records, as applicable.
- **Elimination of less important columns:** The columns that do not contribute any information for predicting the model are eliminated; ’Unnamed: 7’ and ’Unnamed: 8’ are two such columns.
- **Label encoding:** The target variable Output (S) will be encoded to express the fault and normal conditions.
- **Data splitting:** The dataset was split into 70% for training and 30% for testing. This ensures an adequate number of data points for both model validation and training.
- **Normalization:** StandardScaler from scikit-learn was applied for feature normalization on the feature dataset (Ia, Ib, Ic, Va, Vb, Vc). This standardization ensures that the features have a mean of 0 and a standard deviation of 1, which accelerates model convergence during the training process.

**Fig. 2:**
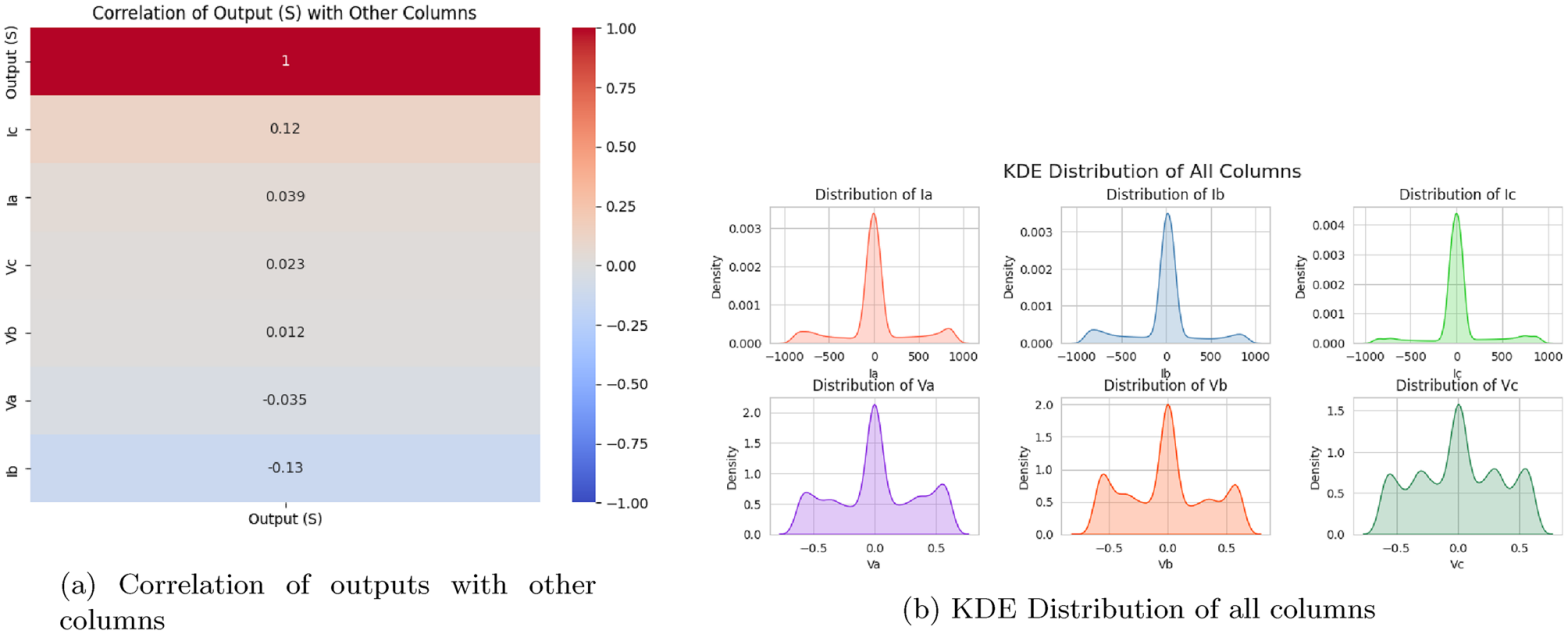
Visualization of data properties.

**Fig. 3:**
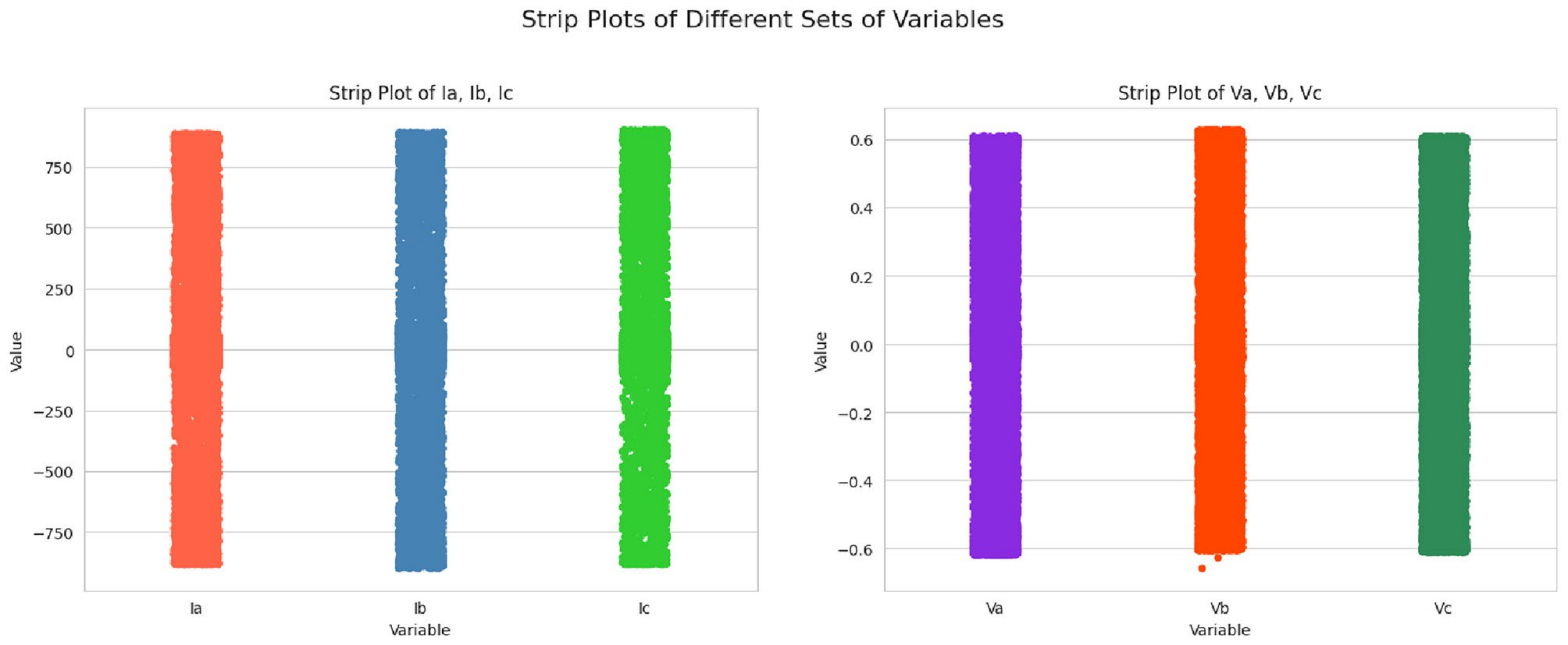
Strip plots of different sets of variables.

**Fig. 4:**
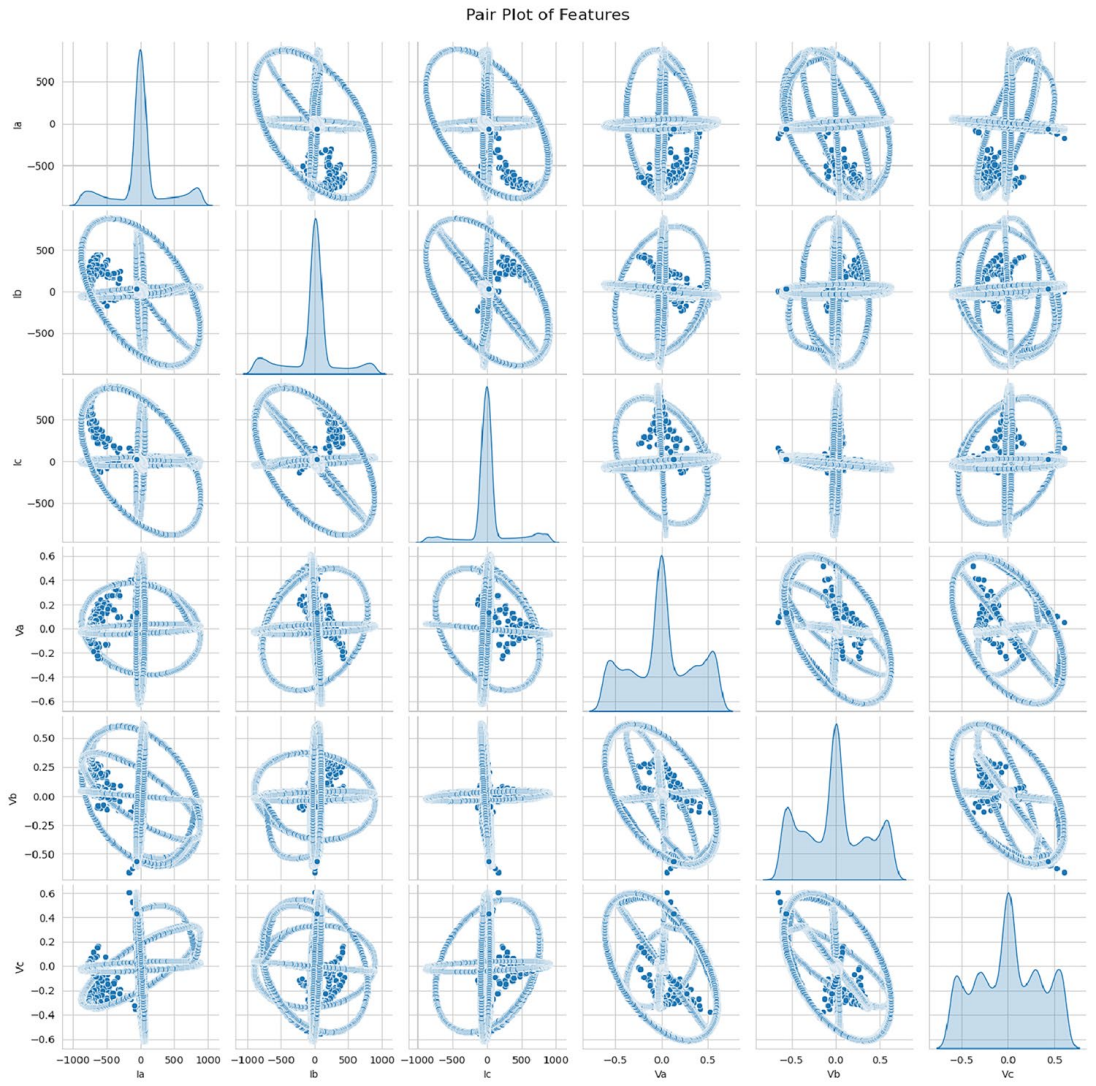
Pair plot of features.

## Preprocessing

Before training the AGFC-Net model, the raw data is preprocessed to ensure it is suitable for machine learning tasks. The dataset consists of voltage and current measurements from various phases of a three-phase transmission line under both normal and fault conditions. The preprocessing steps begin with data cleaning, where any missing or anomalous data points are identified and handled. Outliers are removed to avoid skewing the model’s performance. The raw voltage and current values are then normalized to a consistent range, ensuring that the features are on the same scale and improving the convergence during training. This is particularly important for deep learning models like AGFC-Net, as normalization helps to prevent issues such as vanishing or exploding gradients. Additionally, the data is split into three subsets: a training set (70%), a validation set (15%), and a test set (15%) to allow for model evaluation and tuning. The training set is used to train the model, the validation set is used to optimize hyperparameters and prevent overfitting, and the test set is used to evaluate the final model’s performance. By performing these preprocessing steps, we ensure that the dataset is clean, normalized, and ready for training, enabling the AGFC-Net model to learn more effectively from the data.

## Feature engineering

**Fig. 5:**
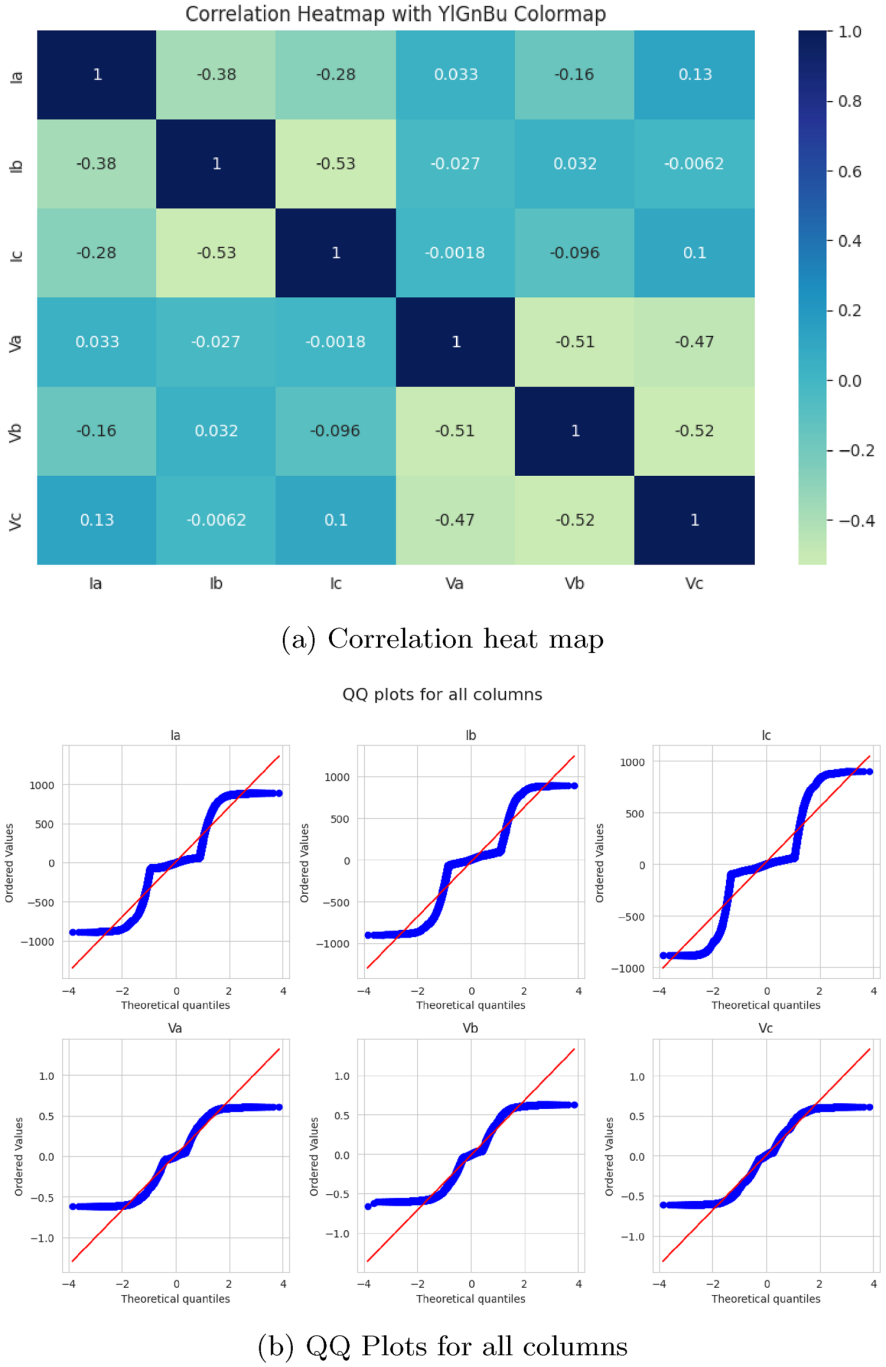
Feature distribution visualizations.

Six input features have direct feeds into the model, and no feature engineering is done above that. Now, those visualizations to get a better view of the feature distributions and their relationships with the target variable are correlation heatmaps (Fig. 2), KDEs (Fig. 3), and QQ plots (Fig. 4, Fig. 5). We can determine the strength of linear relationships between inputs and the target through correlation analysis; therefore, it helps decide how to retain all six features. Furthermore, we will use the QQ plot to check the normality of feature distributions so that features are proper for the model, particularly when linearity or normality is of more advantage for training.

## Proposed model

### Architecture

The suggested architecture of the proposed model (you can refer Fig. 1), the AGFC-Net, The basic core consists of:

- **Convolutional layers**: Two 1D Convolutional layers extract spatial features from sequences of input measurements of current and voltage. These layers have a kernel size of 3 and zero padding to keep the length of the sequence constant.
  The 1D convolution operation is defined as:
  $$\begin{aligned} \text {Conv1D}(x)_i = \sigma \left( \sum _{k=1}^{K} w_k \cdot x_{i+k-1} + b\right) \end{aligned}$$
  where $x$ is the input sequence, $w$ and $b$ are the weights and biases of the convolutional filter, $K$ is the kernel size, and $\sigma$ is the activation function (LeakyReLU).
- **Inclusion of Attention Mechanism**: An attention layer is incorporated within the model to facilitate dynamic feature prioritization during training. The process gives different input features different levels of importance by calculating attention weights. The weights are obtained through a linear transformation of the feature embeddings, followed by softmax normalization over the scores. This causes the model to give increased importance to the relevant features in context, thus improving its discriminative power.
  The attention mechanism is given by:
  $$\begin{aligned} \text {Attention}(x) = \text {Softmax}(W_{\text {attn}} \cdot x + b_{\text {attn}}) \end{aligned}$$
  where $W_{\text {attn}}$ and $b_{\text {attn}}$ are the weights and biases for the attention layer, and the softmax function is used to normalize the attention weights.
- **GRU layer**: A GRU layer is applied to capture sequential dependencies in the data, helping the model capture temporal features that may signify faults in the system. The GRU update rules are as follows:
  $$\begin{aligned} z_t= & \sigma (W_z \cdot [h_{t-1}, x_t])\\ r_t= & \sigma (W_r \cdot [h_{t-1}, x_t])\\ \tilde{h}_t= & \tanh (W_h \cdot [r_t \odot h_{t-1}, x_t])\\ h_t= & (1 - z_t) \odot h_{t-1} + z_t \odot \tilde{h}_t \end{aligned}$$
  where $z_t$ and $r_t$ are the update and reset gates, $\tilde{h}_t$ is the candidate hidden state, $h_t$ is the new hidden state, and $\odot$ denotes element-wise multiplication.
- **Fully connected layers**: The output from the GRU layer is passed through several fully connected layers with LeakyReLU activation, which reduces the dimension of the output to ultimately predict the fault condition. These layers are given by:
  $$\begin{aligned} \text {FC}_1(y)= & \sigma (W_1 \cdot y + b_1)\\ \text {FC}_2(y)= & \sigma (W_2 \cdot y + b_2)\\ \text {Output}(y)= & \sigma (W_3 \cdot y + b_3) \end{aligned}$$
  where $W_i$ and $b_i$ are the weights and biases for the fully connected layers, and $\sigma$ is the LeakyReLU activation function for intermediate layers and sigmoid activation function for the output layer.
- **Regularization**: Batch normalization and dropout techniques are applied to prevent overfitting and help the network generalize well. The batch normalization operation is defined as:
  $$\begin{aligned} \text {BN}(x) = \frac{x - \mu }{\sqrt{\sigma ^2 + \epsilon }} \end{aligned}$$
  where $\mu$ and $\sigma ^2$ are the mean and variance of the batch, and $\epsilon$ is a small constant. Dropout is applied as:
  $$\begin{aligned} \text {Dropout}(x) = x \cdot \text {mask} \end{aligned}$$
  Where the mask is a binary vector sampled from a Bernoulli distribution.
- **Binary classification**: The final output layer uses a sigmoid activation function to generate probabilities between 0 and 1, representing the likelihood of a fault occurring in the system:
  $$\begin{aligned} \text {Output}(y) = \frac{1}{1 + e^{-y}} \end{aligned}$$

### Working

The AGFC model works by first applying convolutional layers, conv1 and conv2, to extract important features from the input data, which consists of voltage and current measurements. These layers automatically learn relevant patterns in the data, with batch normalization applied after each convolutional layer to stabilize training. Regularization is applied using dropout layers to prevent overfitting, ensuring the model generalizes well to unseen data. It then applies the attention mechanism (attnlayer), allowing the model to focus on the most important features by assigning attention weights, which help the model filter out the noise and select relevant information for fault detection. The GRU layer finally captures sequential dependencies in the data, thus enabling the model to detect temporal patterns and dynamic fault behaviors. These techniques combined enable the AGFC model to efficiently and accurately detect faults with improved performance on both accuracy and processing time.

## Results

### Model performance

**Fig. 6:**
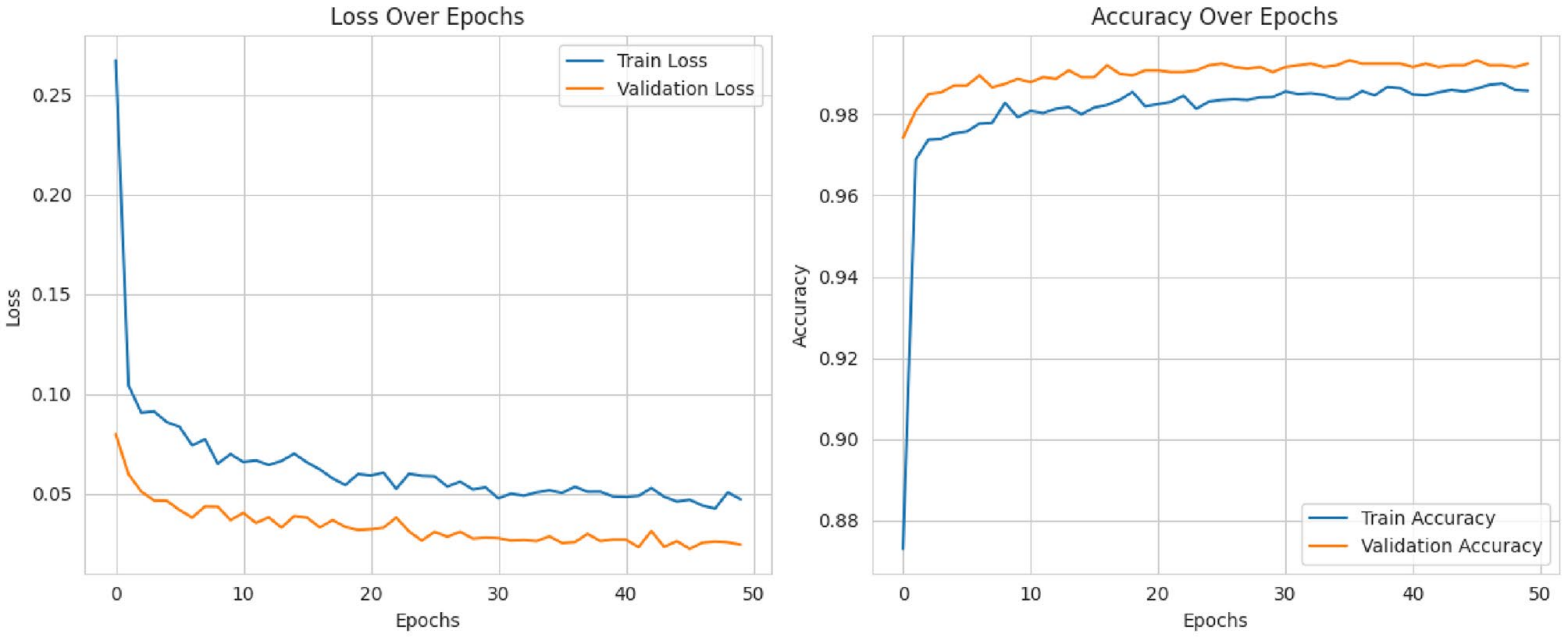
Accuracy and loss plot.

The model proposed here performed exceptionally well, as attested by its training and validation metrics. More than 50 epochs have led to the highly effective reduction of training loss-which does reach stabilization around 0.02-after a rapid increase at the very beginning. Similarly, there was a trend in validation loss, which stabilized at around 0.03. This proves that the learning is effective, showing strong generalization to unseen data. Actually, for the first epochs, training accuracy had risen very well, achieving more than 98% by the 10th epoch and 99.52% at the end of the training. The validation accuracy corresponded to the training one and reached 99.52% as well. Both loss and accuracy metrics (Fig. 6) convergence proves that the model had successfully avoided those situations where it overfitted or underfitted the data and thus was making reliable and stable predictions. The results further confirm the fact that this model is highly capable and persistently performs well on the training dataset and the validation set.

### Confusion matrix

**Fig. 7:**
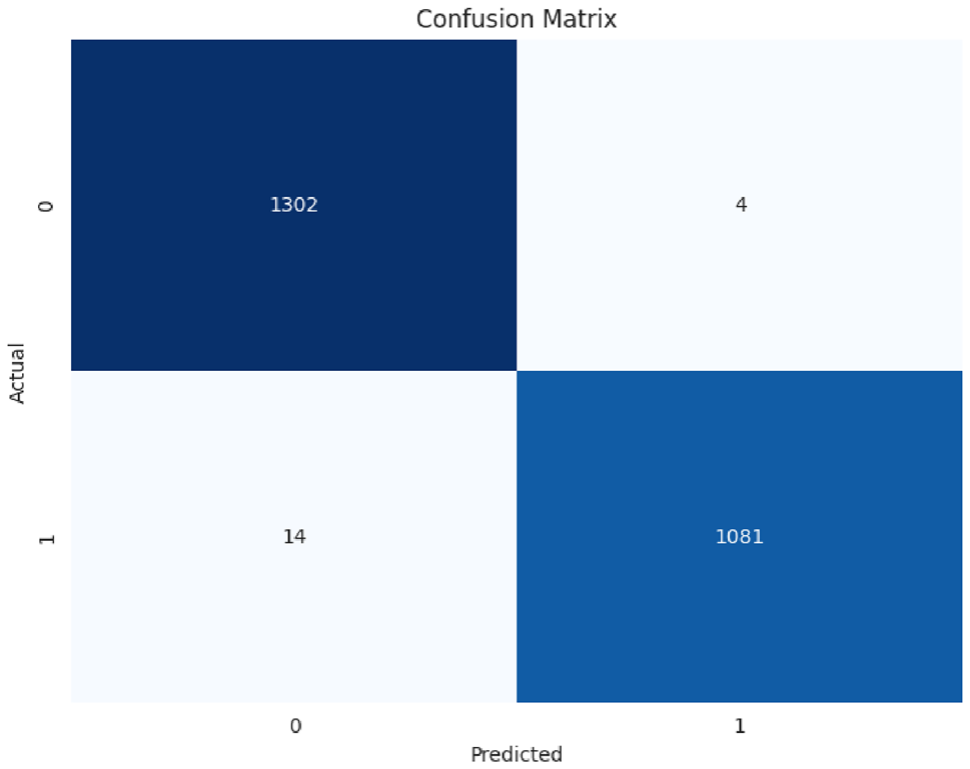
Confusion matrix.

The confusion matrix in Fig. 7 indicates the performance of the model concerning the classification of the test dataset. The model correctly classified 1302 as class 0 and 1081 as class 1, thus showing good ability in differentiation between the classes. This is the least false positives of 4 class 0 samples being treated as class 1, and only 14 instances of class 1 being reported as class 0. These results suggest extremely balanced quality between sensitivity and specificity with little misclassification. In addition, the high number of correctly classified samples along with low errors further confirms the robustness and reliability of the model. This performance is in line with the overall accuracy of 99.52% discussed above.

### Classification report

The classification report evaluates the performance of a classification model by reporting key metrics for each class: precision, recall, F1-score, and support shown in Table 2. Precision is given as the fraction of correctly classified positive samples out of all those classified as positive, and recall is the fraction of actual positive samples identified. The F1-score is the harmonic mean of precision and recall. High scores were reflected in both classes because precision, recall, and F1-scores stood at above 0.99; hence, the classification would prove to be balanced and accurate. The number of support column points of real samples in each class indicates that the dataset generally maintains balance. With 1306 samples for Class 0 and 1095 for Class 1, the overall accuracy of the model stands at 99.54%, with macro and weighted averages suggesting consistency across classes. These metrics collectively demonstrate that the model is strong in its classification capabilities.

**Table 2:** Classification report with confidence interval and statistical metrics.

Class	Precision	Recall	F1-Score	Support
0	0.99	1.00	0.99	1306
1	1.00	0.99	0.99	1095
**Accuracy**			0.99	2401
Macro Avg	0.99	0.99	0.99	2401
Weighted Avg	0.99	0.99	0.99	2401
**Additional Metrics**				
Accuracy CI (95%)		(0.9896, 0.9963)		
Prediction Variance		0.2477		
Ground Truth Variance		0.2481		
Chi-squared Test p-value		$0.0000 \times 10^{0}$		

### Comparison with other models

Comparison of various models for classification shows significant differences in accuracy Table 3. Traditional machine learning models like SVM (99.21%) and ANN (97.92%) are proven to be highly efficient, but they are slightly beaten by deep learning-based models like LSTM-DWT (99.04%) and DWT-BPNN (97.22%). Although LSTM-based models are typically good at capturing sequential dependencies, the performance of these models is unpredictable, as seen in the case of Veerasamy’s LSTM model that resulted in a lower accuracy of 92.42%. Conversely, the highest accuracy of 99.52% is attained by the proposed Attention-GRU-Based Fault Classifier (AGFC-Net) outperforming all tested models. The uniqueness of AGFC-Net is that it combines an attention mechanism with GRU architecture, allowing the model to learn to selectively attend to the most important temporal features without the inclusion of irrelevant information. This focused attention facilitates more efficient extraction of complex fault-related patterns and enhances robustness against noisy data. In contrast to more complicated hybrid structures like CNN-LSTM or Transformer models, AGFC-Net attains better performance while requiring less computational complexity, thereby being well suited for real-time industrial applications. Its capacity for combining interpretability, efficiency, and accuracy puts AGFC-Net in an extremely effective and scalable fault classification solution.

**Table 3:** Comparison of models and their accuracy.

Author	Model	Accuracy
M. Z. Yousaf	LSTM-DWT	99.04
Veerasamy	LSTM	92.42
C. Pothisarn	DWT-BPNN	97.22
B. Samanta	ANN	97.92
P. Ray	SVM	99.21
**Proposed Model**	**AGFC**	**99.52**

## Conclusion and future scope

In this paper, we proposed a model called AGFC-Net, a classifier based on attention-GRU for effective fault detection in electrical power systems. The proposed model utilized convolutional layers that extract spatial features and apply the attention mechanism to find relevant features. GRU layers are used further to capture sequential dependency. Fully connected layers combined with techniques of batch normalization and dropout helped refine features and avoid overfitting, respectively. As the technique was for the binary classification approach, the model could easily make effective predictions regarding fault conditions.

The proposed experimental results confirm that AGFC-Net can potentially detect faults in power systems if the input measurements of current and voltage are fed to the model. The integration of attention mechanisms and GRU layers into the model enhances its spatial features as well as temporal features, significantly improving its fault detection ability.

### Future scope and limitations

Despite the promising results obtained from the proposed AGFC-Net, several avenues for further exploration exist:

- **Application to multiclass fault detection:** Future work may involve the development of a multiclass fault-classification model, which would allow the classification of various types of faults in the power system beyond binary classification.
- **Incorporation of additional features:** The model can be extended by incorporating additional features such as environmental conditions, equipment health data, or other electrical parameters that might improve the accuracy of fault detection and classification.
- **Real-time fault detection:** Implementing the AGFC-Net in real-time fault detection systems can provide practical benefits by enabling immediate responses to faults, thereby preventing damage to the power system and reducing downtime.
- **Optimization of model architecture:** Further optimization of the model architecture, including experimenting with different types and combinations of neural network layers, may lead to performance and efficiency improvements, especially for large-scale systems.
- **Transfer learning and domain adaptation:** Exploring the applicability of transfer learning techniques and domain adaptation could allow the model to be used in different power systems with unique configurations and operating conditions, thus improving its generalization capabilities across varied environments.

However, there are several limitations associated with the AGFC-Net model that must be considered:

- ** Dependence on labeled data:** The model’s performance is highly dependent on the availability of labeled data, which might not always be readily available in real-world scenarios. Labeling large datasets can be time-consuming and expensive, and without sufficient labeled data, the model’s ability to generalize may be hindered.
- ** Limited generalization to unseen fault types:** While the model performs well on the faults in the training dataset, its generalization to unseen fault types or scenarios not present in the dataset remains a challenge. Future work should focus on improving the model’s robustness to new and diverse fault patterns.
- ** Real-time processing challenges:** Although the model is designed for faster processing times, real-time fault detection in large-scale power systems with high-frequency data may still pose significant computational challenges. Optimizing the model to handle high-speed data in real time is a crucial area for future improvement.
- **Scalability in large networks:** The model’s scalability to handle very large power systems, with complex interconnections and a large number of faults, remains an open issue. There is a need for methods that enable efficient processing and fault detection in extensive networks with limited computational resources.
- **Sensitivity to data quality:** The AGFC-Net model’s performance can be significantly impacted by poor-quality data, such as noisy measurements or missing values. While preprocessing techniques help mitigate this, the model’s reliability is still affected by the quality of the data it receives.

## Data Availability

No data was generated during the study.
